# How to teach otologic surgery to the next generation?

**DOI:** 10.1016/j.bjorl.2025.101749

**Published:** 2026-01-17

**Authors:** Amanda Sampaio Almeida, José Ricardo Gurgel Testa

**Affiliations:** Universidade Federal de São Paulo (UNIFESP), Escola Paulista de Medicina, Department of Otolaryngology-Head and Neck Surgery, São Paulo, SP, Brazil

## Introduction

The advent of new technologies in medicine, coupled with changes in undergraduate and medical residency curricula, demands that the centuries-old approach to teaching surgical skills be adapted to a new generation.

Within this context, the scope of Otorhinolaryngology has expanded, with various subspecialties defined as prerequisites for specialist medical training. Among them, Otology is recognized as one of the most traditional and technically complex. Its intricate anatomy, involving delicate structures and multiple surgical approaches—such as endoscopic vs. microscopic or combined techniques, retroauricular vs. transcanal vs. lateral skull base access — presents significant challenges during the learning process. Additional barriers include the reduced availability of cadaveric dissection and the high costs of alternative simulation methods for the temporal bone.^1^

This editorial aims to identify training strategies suited to the new generation and methods that optimize the surgical learning curve.

## Pillars of otologic training: anatomical knowledge, hands-on practice and supervised training

Anatomical knowledge is fundamental to acquiring surgical skills, particularly in such a complex area. A detailed understanding of temporal bone and middle ear anatomy is essential for recognizing structures, developing the ability to plan surgical techniques and minimizing errors and complications. Traditional teaching methods rely on two-dimensional images from books and online resources, which are limited in conveying the depth of temporal bone. In contrast, the use of endoscopes has gained popularity as an educational tool, offering wide and dynamic visualization of the anatomy- including recesses and sinuses — advantages frequently cited by otolaryngology trainees.

Hands-on training currently involves two main approaches: cadaveric and synthetic models. Cadaveric specimens offer a unique opportunity for trainees to develop tactile skills in temporal bone drilling, as well as to identify anatomical variations. These experiences prepare the trainee for real-world scenarios, thereby, enhancing patient safety and surgical outcomes. However, ethical, logistical, and financial constraints have limited cadaveric training, prompting the development of synthetic models. Three-dimensional (3D) printed temporal bones have emerged as viable alternatives, with resin being the preferred material. These models allow anatomical customization and are more cost-effective than other options. Nevertheless, soft tissue structures like the facial nerve remain challenging to replicate accurately. Additional limitations include poor simulation of bleeding, lack of standardization, and environmental concerns related to 3D printing.

Supervised surgical training relies on the concept of the learning curve, whereby skills improve progressively with time and practice. The introduction of endoscopic ear surgery — a milestone in modern otology — presents new technical and pedagogical challenges. This approach demands unique skills such as single-handed technique, bleeding management and adaptation to reduced depth perception. Despite advancements and growing acceptance, endoscopic surgery still faces resistance from some traditionally trained otologists. This is often attributed to the steep learing curve associated with mastering a new technique after becoming proficient in the conventional microscopic approach. Such resistance highlights the need for innovative teaching methodologies.

## Teaching methodologies

The classic “see one, do one, teach one” model proposed by Halsted dominated surgical education for nearly a century but has been increasingly criticized for safety concerns and insufficient student engagement. In contrast, Walker and Peyton (1998) proposed a four-step method for procedural training.^2^ The steps consist of: demonstration – the supervisor performs the entire procedure; deconstruction – the teacher repeats the demonstration describing the steps of the process; comprehension, – the student describes the procedure and ``guides`` the teacher during its execution; and execution – the student performs the procedure with feedback from the teacher.

Another promising model is "Intelligent Cooperation," which promotes active intraoperative teaching through verbal and physical interaction. The assistant prompts the trainee with questions about next steps, topographic anatomy, and instruments, encouraging equal participation. This model allows precise identification of difficulties and timely corrective feedback while emphasizing the cooperative nature of surgical education.^3^

Regarding the learnig curve, there are four key factors which influence.^4^ The first is the content complexity, which dictates the time needed for mastery. Second one is the teaching method, which depends on the instructor's pedagogical approach; A third fator is the practice frequency, which reinforces learning through repeated exposure; Finally, is the motivation, which is driven both intrinsically by student engagement and extrinsically by the learning environment.

Modern learners require tailored strategies to optimize their learning curves. Pedagogical concepts such as microlearning, adaptive learning, gamification, and continuous feedback have shown promise. Microlearning breaks down skills into smaller tasks. Adaptive learning adjusts to the learner’s needs by identifying weaknesses. Gamification incorporates game elements such as points and rewards to increase engagement. Continuous feedback provides ongoing assessment to drive improvement. Millennials and Generation Z, known for multitasking, technological fluency, and shorter attention spans, benefit from those active and dynamic learning methods.^5^

To modernize otologic surgical education, early exposure to both endoscopic and microscopic training is essential. Residents reveal optimism about the educational value of endoscopy, highlighting its enhanced visualization. Specialits increasingly support a complementary approach, advocating proficiency in both techniques for the modern otologist.

Future research should focus on how to assess trainee performance. Traditionally, the learning curve has been evaluated by surgical volume and operative time. However, skill acquisition and competency should be prioritized over case numbers. Proposed alternative metrics include rates of success, such as audiological outcomes and tympanic membrane closure, number of attempts per surgical step and complication rates. New assessment tools should be standardized to advance surgical education.

## Conclusion

Otologic surgical education must incorporate a diverse array of teaching tools, integrating cadaveric dissection, virtual technologies, and supervised operative training. Balancing theory with hands-on experience is crucial to ensure a safe and progressive learning. The traditional model based solely on case volume should be replaced by competency-based assessment, focusing on technical skills and defined learning objectives. Future studies should investigate standardized evaluation tools, curriculum development and the perceptions of both trainees and educators to ensure the continued evolution of otologic education.

## ORCID ID

Amanda Almeida: 0000-0002-1496-6258

José Ricardo Gurgel Testa: 0000-0001-9632-738X

Authors’ contributions

Amanda Sampaio Almeida: Literature review, data collection, study`s design and writing.

José Ricardo Gurgel Testa: Literature review, data collection, study`s design and writing.

All authors read and approved the final manuscript.

## Conflicts of interest

The authors declare no conflicts of interest.

## Data availability statement

The authors declare that all data are available in repository.
